# Expression Patterns of miRNAs in Egyptian Children with ADHD: Clinical Study with Correlation Analysis

**DOI:** 10.1007/s12031-024-02220-8

**Published:** 2024-04-23

**Authors:** Hala M. Zeidan, Neveen Hassan Nashaat, Maha Hemimi, Adel F. Hashish, Amal Elsaeid, Nagwa Abd EL-Ghaffar, Suzette I. Helal, Nagwa A. Meguid

**Affiliations:** 1 Research on Children with Special Needs Department, Medical Research and Clinical Studies Institute, National Research Centre, El-Buhouth St., Dokki 12622, Cairo, Egypt; 2 Clinical and Chemical Pathology Department, Medical Research and Clinical Studies Institute, National Research Centre, El-Buhouth St., Dokki 12622, Cairo, Egypt

**Keywords:** ADHD, microRNA, BDNF, Children, Cognition, Gender

## Abstract

ADHD has huge knowledge gaps concerning its etiology. MicroRNAs (miRNAs) provide promising diagnostic biomarkers of human pathophysiology and may be a novel therapeutic option. The aim was to investigate the levels of miR-34c-3p, miR-155, miR-138-1, miR-296-5p, and plasma brain-derived neurotrophic factor (BDNF) in a group of children with ADHD compared to neurotypicals and to explore correlations between these measures and some clinical data. The participants were children with ADHD in Group I (*N* = 41; age: 8.2 ± 2) and neurotypical ones in Group II (*N* = 40; age: 8.6 ± 2.5). Group I was subjected to clinical examination, the Stanford Binet intelligence scale-5, the preschool language scale, and Conner’s parent rating scale-R. Measuring the expression levels of the miRNAs was performed by *qRT-PCR* for all participants. The BDNF level was measured by ELISA. The lowest scores on the IQ subtest were knowledge and working memory. No discrepancies were noticed between the receptive and expressive language ages. The highest scores on the Conner’s scale were those for cognitive problems. Participants with ADHD exhibited higher plasma BDNF levels compared to controls (*p* = 0.0003). Expression patterns of only miR-34c-3p and miR-138-1 were downregulated with significant statistical differences (p˂0.01). However, expression levels of miR-296-5p showed negative correlation with the total scores of IQ (*p* = 0.03). MiR-34c-3p, miR-138-1, while BDNF showed good diagnostic potential. The downregulated levels of miR-34c-3p and miR-138-1, together with high BDNF levels, are suggested to be involved in the etiology of ADHD in Egyptian children. Gender differences influenced the expression patterns of miRNAs only in children with ADHD.

## Introduction

The prevalence of attention deficit hyperactivity (ADHD) in worldwide population is estimated to be 5.9% among youths and 2.5% among adults (Faraone et al. 2021). Diagnosis of ADHD is challenging due to missing gaps related to knowledge of its etiology (Dypås et al. 2024). Individuals with ADHD were reported to have differences in their brain processes, which produce changes in personal, social, academic, and occupational functioning (American Psychiatric Association 2022). The etiology of ADHD is still poorly recognized. However, there are indications of genetic and environmental factors contributing to this disorder (Koutsoklenis et al. 2023). On the other hand, epigenetic changes, especially microRNAs, have been suggested to be involved in factors that govern the progress of ADHD.

MiRNAs are non-coding small RNAs (19- to 24-nucleotides) that regulate gene expression post-transcriptionally. Moreover, they bind to the 3-untranslated region of the target messenger RNAs (mRNAs). Their role is suggested to inhibit mRNA translation or its degradation, acting as positive regulatory factor (Garcia-Martínez et al. 2016). Many miRNAs participate in synaptic plasticity, synaptogenesis, cell proliferation, cell differentiation, and apoptosis in the central nervous system (Wang et al. 2022b). Growing evidence for miRNAs has been suggested to play an important role serving as potential therapeutic targets for ADHD treatment (Kandemir et al. 2014; Srivastav et al. 2018).

Expression levels of many types of miRNAs were investigated in individuals with ADHD and were found to be dysregulated. These, includes miR-34c-3p, miR-155, miR-138-1, and miR-296-5p, miR-140-3p, miR-126-5p, miR-4516, miR-6090, miR-4763-3p, miR-4281, miR-4466, miR-107, miR-183-96-182, miR-641, miR-101-3p, miR-130a-3p, miR-195-5p, and miR-106b-5p (Garcia-Martínez et al. 2016; Martinez and Peplow 2024; Zadehbagheri et al. 2019). Furthermore, the genes controlled by these miRNAs were involved in their mechanism of action and were reported to be altered in children with ADHD (Srivastav et al. 2018).

The miR-34 family specifically participates in stem cell differentiation, neuronal development, aging, and some metabolic functions. MiR-34c was reported to be downregulated in the amygdala, substantia nigra, and frontal cortex in Parkinson’s disease, where it was linked to Alzheimer’s disease, anxiety, or preclinical manifestations of Huntington’s disease (Garcia-Martínez et al. 2016). It was reported that aberrant expression of miR-34b and miR-34c in the peripheral blood of subjects with autism spectrum disorder (ASD), was linked to ADHD (Garcia-Martínez et al. 2016). On the other hand, miR-155a-5p levels were reported by Kandemir et al. (2014) to be upregulated in children with ADHD compared to neurotypical children. MiR-138 was reported to inhibit proliferation inducing apoptosis; it is furtherly identified as having a negative correlation between its expression and DNA methylation (Sha et al. 2017).

Brain-derived neurotrophic factor (BDNF) was reported to be involved in cellular growth and neuronal differentiation in addition to synaptic plasticity, especially in brain areas involved in memory and learning. Both miR-138-1 and miR-296-5p were reported to be involved in controlling the BDNF pathway and its mechanism of action (Wu et al. 2017). BDNF has been reported to be altered in ADHD and learning disorders. High level of BDNF was reported in children with ADHD (El Ghamry et al. 2021; Gumus et al. 2022), and reduced in children with learning disorders (Elhadidy et al. 2019). On the other hand, some studies did not detect alterations in BDNF level compared to neurotypical individuals (Bilgiç et al. 2017; Scassellati et al. 2014). The involvement of BDNF in the control of neuronal development and survival is complicated. Some brain neurotransmitters are interrelated with this neurotrophic factor (Bathina and Das 2015). Moreover, it was reported to be involved in the etiology of ADHD (Liu et al. 2015).

Children with ADHD exhibited different cognitive discrepancies even when the total intelligence quotient (IQ) is within the normal range. Verbal communication was one of the major affected cognitive functions affected in these children. On the other hand, language development has an essential function in overall cognitive and social progress. Many children with ADHD were reported to manifest delayed language development (Sciberras et al. 2014). Children with ADHD with comorbid language impairments usually manifested verbal and semantic fluency disorders (Kilany et al. 2022). Where some of them exhibited limited utterance, reduced syntactic complexity, and phonological errors (Kim and Kaiser 2000). There is an overlap between language disorder and language delay in children with ADHD. Therefore, identifying their performance in receptive and expressive language abilities is essential for determining points of weakness and strength in their abilities, in addition to the nature of the associated language deficits (Méndez-Freije et al. 2024). They could manifest delays in executive function performance and working memory tasks (Gremillion and Martel 2012). These deficits were related to alterations in the levels of essential elements that are involved in neuronal development and differentiation, such as miRNAs, and BDNF (Martinez and Peplow 2024). To our knowledge, none of these miRNAs have been previously investigated in Egyptian children with ADHD.

The aim of this study was to investigate the levels of miR-34c-3p, miR-155, miR-138-1, and miR-296-5p in a group of children with ADHD compared to neurotypical children. Estimating the levels of BDNF and exploring possible links between the miRNAs expression patterns, BDNF levels, and some clinical measures were targeted.

## Methods

The study included 81 children: Group I for children with ADHD (*N* = 41; age: 8.2 ± 2; 34 males, 7 females) and Group II for neurotypical children (age: 8.6 ± 2.5; 30 males, 10 females). The inclusion criteria for group I was the diagnosis of ADHD according to the criteria of the DSM-5 (American Psychiatric Association 2013), and their age ranged from 6 to 12 years. The diagnosis was performed via a semi-structured interview by a psychiatrist at the time they were included in the study. Children were visiting the outpatient clinic of the research on children with special needs department at the Medical Research Centre of Excellence, National Research Centre, in the years 2021 and 2022. Children with comorbidities such as ASD, intellectual disability, developmental coordination disorder, and chronic illnesses, in addition to those with features suggestive of syndrome involvement, delays in gross motor development, or those who were receiving medications, were excluded from the study. The motor development was checked by a physician based on the normal developmental charts in pediatrics. Comorbid psychiatric disorders were excluded by a psychiatrist using a mini-international neuropsychiatric interview for children and adolescents (Ghanem et al. 2000). For Group II, children were included when their age ranged from 6 to 12 years and they were performing well in their schools. Children with a history of delayed language or motor development were excluded. They were volunteers who agreed to join the study. They were subjected to history-taking and clinical examination, which included verifying their proper motor and language development by a physician. Children in Group I were subjected to history-taking and clinical examination, including general, neurological, otorhinolaryngological, and phoniatric exanimation. The Stanford Binet intelligence scale, fifth edition (Farag 2013), was performed to get the intelligence quotients (IQ) of the participants by a skilled health professional in psychometry. The preschool language scale was performed to obtain language age scores by a phoniatrician. The ceilings of the preschool language scale regarding the receptive language, expressive language, and total language ages were 6.58, 7, and 6.9, respectively. This scale measures the language performance regarding these 3 parameters from the age of 2 months to the age of 7.5 years. When children older than 7.5 years obtained the highest scores of the scale, they were given the scores of the test ceiling, and their language performance was considered to be fully developed according to the test manual. The scaled scores were used to determine the presence or absence of an associated language delay. Obtaining scaled scores greater than 77.5 in any of the parameters indicated normal language development (El-Sady et al. 2011). The Conners’ Parent Rating Scales-Revised (CPRS-R) was performed to determine the severity of ADHD by expert physicians (Conners 1997).

Venous blood samples (5 ml) were obtained from all participants in RNase-free sterile tubes containing EDTA and placed on ice for RNA extraction. The plasma was separated by centrifugation of the blood sample for 10 min at 1900 xg at 4 °C. The plasma was transferred carefully into new RNase-free, sterile eppendorf tubes. The plasma was centrifuged again for 10 min at 12,000 xg at 4 °C to avoid contamination of cellular nucleic acid. The hemolyzed plasma samples were excluded from the study and re-collected from the patients or controls. The aliquots of the resultant plasma samples were stored at -80 °C until BDNF measurement. The total RNA was immediately extracted from the 200 µl plasma of the participants by using the miRNeasy serum/plasma cell lysate kit (Qiagen, Germany), according to the manufacturer’s instructions. The RNA purity and integrity were screened by using a nanodrop spectrophotometer (2000 c, Thermoscientific, USA) and kept at -80 °C until further processing. Reverse-transcription was carried out for cDNA synthesis by using a miScript HiSpec buffer supplied in the miScript II RT Kit (Qiagen, Germany), following the supplier’s instructions. The expression profiles of the selected miRNAs were carried out using quantitative real-time polymerase chain reaction (*qRT-PCR*) (DNA Technology, Moscow, Russia) using miScript SYBR Green PCR master mix (Qiagen, Germany) with miScript primer assays (Qiagen, Germany) specific for the selected miR-138-1, miR-296-5p, miR-34c-3p, and miR-155. MiR-16 was used as the endogenous control (Huang et al. 2010). The reaction mixture was carried out in a total volume of 20 µl containing 4 µl of cDNA (100 ng/µl). A quantity of 300 nM of each primer, 10 µl of SYBR Green Master Mix, and nuclease-free water were used to complete the total reaction volume up to 20 µl. The thermal cycling profile was initial denaturing at 95 °C for 15 min, followed by 40 cycles of 95 °C for 30 s, annealing at 55 °C for 30 s, and extension at 72 °C for 45 s. The miRNAs-fold changes were calculated using the 2^−∆∆ct^ method (Schmittgen and Livak 2008). BDNF levels were measured in plasma samples (Chul et al. 2009) for each participant using the Human Brain Derived Neurotrophic Factor ELISA Kit (SinoGeneClon Biotech, China), following suppliers’ instructions.

Written informed consent was obtained from the parents of each participant. The medical research ethics committee of the National Research Centre approved the study (no. 19355). This cross-sectional study followed the strengthening the reporting of observational studies (STROBE) checklist (Cuschieri 2019).

### Statistical Analysis

Data were analyzed using the Statistical Package for Social Sciences, version 22.0 (SPSS) and GraphPad Prism, version 6. The quantitative data were presented in the form of mean and standard deviation or standard error, and the qualitative data were presented as number and percentage. The numerical datasets were compared by the student t test for parametric data or the non-parametric Mann-Whitney U test. The effect size (Cohen’s d) was calculated. Qualitative variables were compared using the Chi-square test. A two-tailed p-value of ≤ 0.05 was considered statistically significant. The Spearman correlation coefficient test was used for testing correlations. Correlations between the miRNAs expression levels and each other and between miRNAs expression levels and BDNF levels, age, IQ, and CPRS-R subitems, in addition to receptive, expressive, and total language ages, were investigated. The receiver operating characteristic (ROC) curve was plotted to assess the fitness of miR-34, miR-155, miR-138, miR-296-5p, and BDNF as diagnostic biomarkers for ADHD.

## Results

**Fig. 1:**
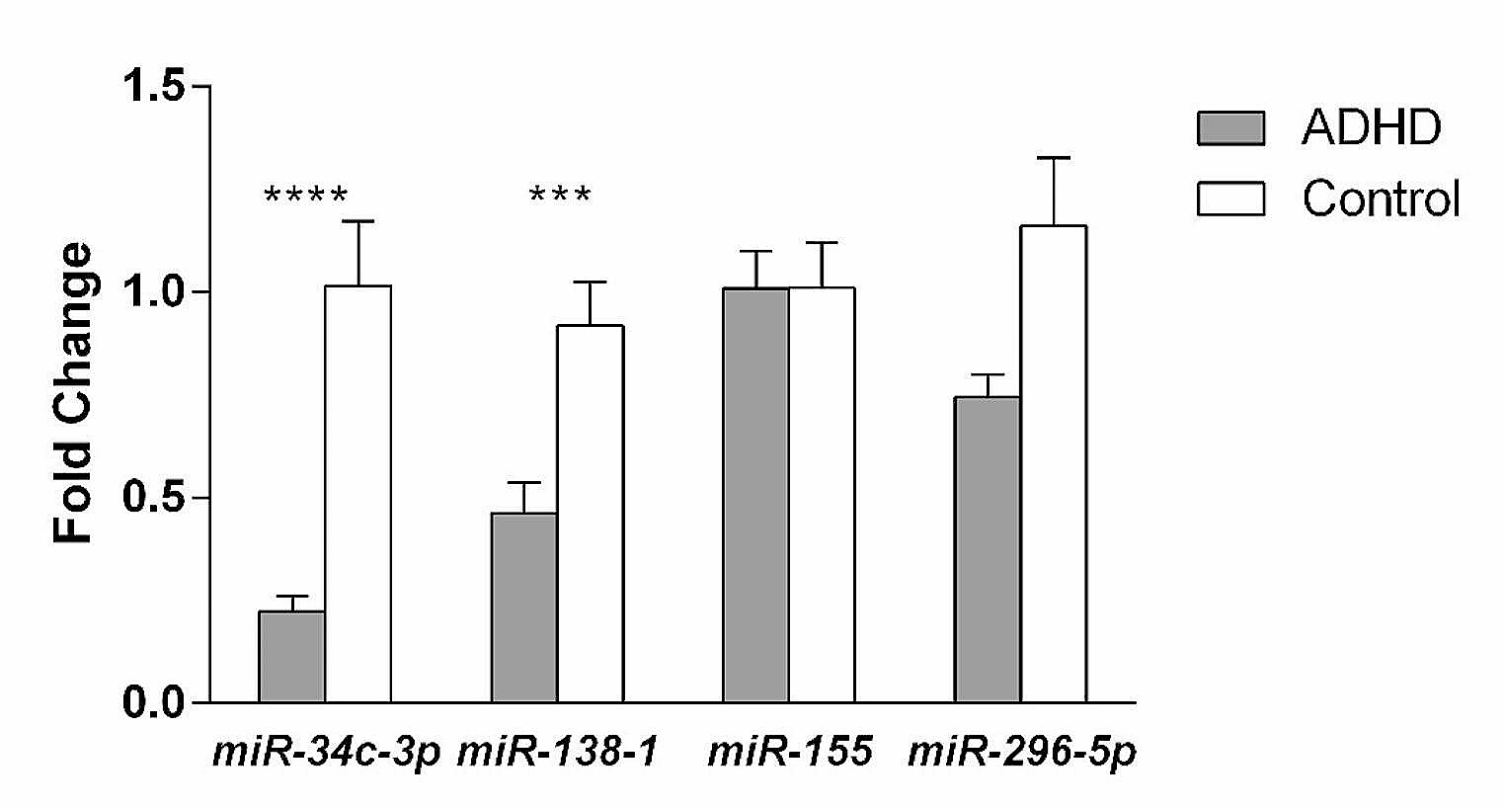
The expression patterns of the four miRNAs in ADHD children compared to neurotypical controls. **A** miR-34c-3p, **B** miR-138-1, **C** miR-155, **D** miR-296-5p. ***: *p* < 0.001; ****: *p* < 0.0001

Comparisons between the two groups regarding age and sex distribution revealed non-significant statistical differences (Table 1). The range of the IQ was 77–130. The lowest scores on the Stanford Binet intelligence scale were those for knowledge and working memory subitems. The highest scores were the quantitative reasoning scores. The ranges of the receptive, expressive, and total language ages were 3.5–6.58; 3–7; and 3-6.9 years, respectively. Children who manifested delays in language development were those with a chronological age less than 8 years (*N* = 14; 34.1%). In these children, the difference between receptive and expressive language ages did not exceed 6 months. The results of the IQ and language scales for Group I are presented in Table 2. The highest scores of the CPRS-R were obtained in cognitive problems, restlessness, social problems, and oppositional traits. The results of the CPRS-R are presented in Table 2. The expression levels of miR-138-1 and miR-34c-3p were significantly downregulated in Group I. The effect size estimation revealed that the d values for these two types of miRNAs were 0.01 and 4.9, respectively, which reflect the strength of using miR-34c-3p in ADHD cases. The miR-155 and miR-296-5p expression patterns did not show significant differences. The patterns of expression of the four types of miRNAs are presented in Table 3; Fig. 1. The BDNF levels were higher in Group I compared to Group II, with significant statistical differences (Table 3). The estimated d value for the BDNF level was 5, which indicated the strength of measuring it in ADHD cases.

Correlation analysis revealed that expression levels of miR-34c-3p were negatively correlated with miR-138-1 (*r* = -0.41, *p* = 0.007). The miR-296-5p showed a negative correlation with the total scores of IQ (*r* = -0.32, *p* = 0.03). No other correlations were detected. In the control group, a significant positive correlation was found between miR-34c-3p and miR-296-5p expression levels (*r* = 0.37; *p* = 0.01). Furthermore, a significant positive correlation between miR-155 and miR-138-1 expression levels has been observed (*r* = 0.35; *p* = 0.02). No correlations were found between the studied miRNAs and the BDNF levels in the ADHD group or the neurotypical children.

**Fig. 2:**
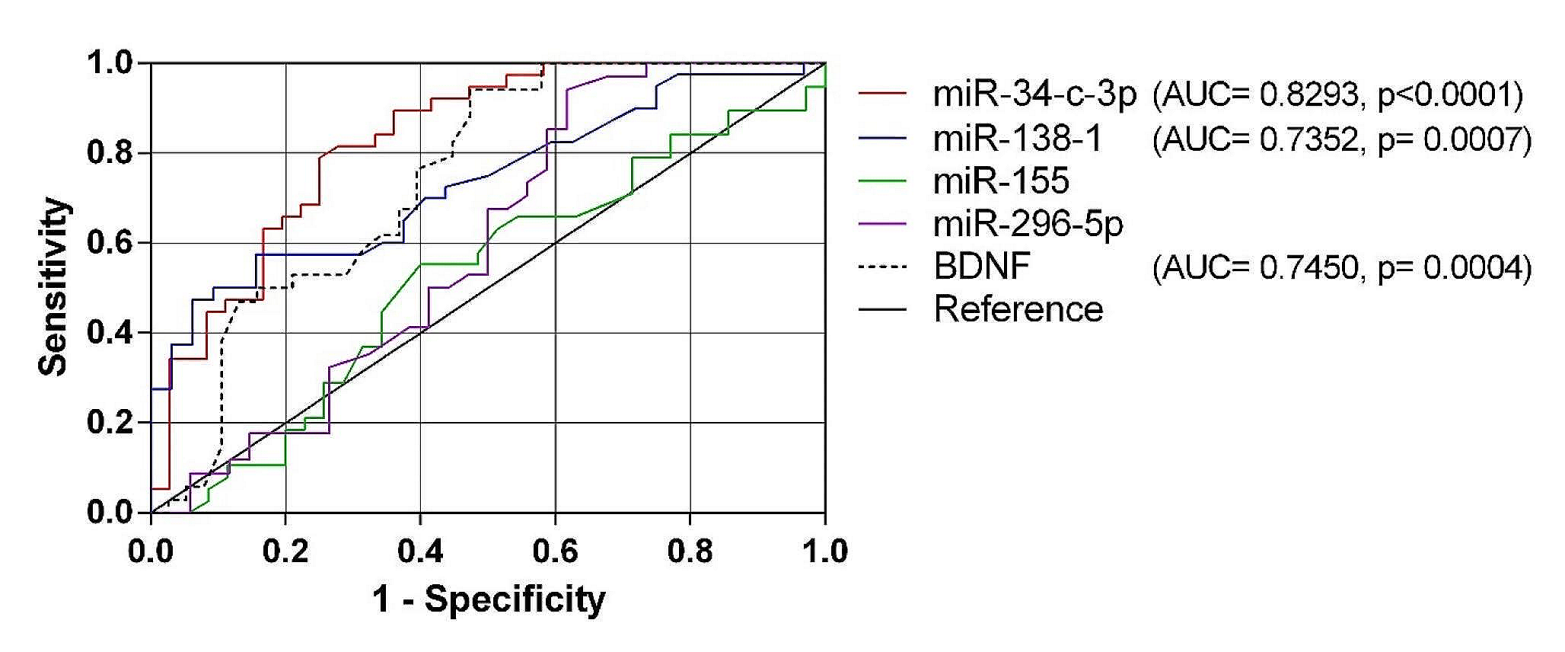
ROC curve of the miRNAs and BDNF levels in children with ADHD

The ROC curve analysis for miR-34c-3p showed excellent discrimination accuracy (AUC = 0.8293, 95% CI = 0.7355 to 0.9231, *p* < 0.0001). The sensitivity and specificity at the cutoff point of 0.4157 were 81.58% and 72.22%, respectively. The miR-138-1 and BDNF showed acceptable discrimination accuracy (AUC = 0.7352, 95% CI = 0.6217 to 0.8486, *p* = 0.0007, and AUC = 0.7450, 95% CI 0.6301 to 0.8598, *p* = 0.0004, respectively). For miR-138-1, the sensitivity and specificity at the cutoff point of 0.8611 were 75.61% and 60%, whereas those for BDNF were 76.47% and 60.53%, respectively, at the cutoff value of 1255ng/µl. The miR-155 and miR-296-5p did not show discrimination between cases and controls (Fig. 2).

The gender differences in the ADHD group concerning the levels of the miRNAs were statistically significant only regarding miR-34c-3p and miR-155 being higher in females, whereas the BDNF levels were lower in females of both ADHD and control groups without a significant statistical difference. These differences concerning miR34c-3p and miR-155 were not detected in the control group (Table 4).

Correlation analysis in males with ADHD revealed that expression levels of miR-34c-3p remained negatively correlated with miR-138-1 (*r* = -0.48, *p* = 0.003). The miR-296-5p showed a correlation with the total scores of IQ in males (*r* = -0.3, *p* = 0.02). Correlations between the levels of expression of miRNAs and each other and with BDNF levels in females with ADHD were non-significant. No correlations were detected between the clinical measures and the levels of the miRNAs or the BDNF levels in males and females with ADHD. In the control group, the correlation between miR-34c-3p and miR-296-5p expression levels remained significant in the male group (*r* = 0.4; *p* = 0.01). The correlation between miR-155 and miR-138-1 expression levels remained significant in the male group (*r* = 0.42; *p* = 0.01). No correlations were detected in the female group.

Comparisons between males in the ADHD group and males in the control group revealed significant statistical differences regarding miR-34c-3p and miR-138-1 being downregulated. The expression of miR-155 was downregulated in males with ADHD without a significant statistical difference. Nevertheless, the expression of miR-155 was upregulated in females with ADHD, with a significant statistical difference contradicting the overall results. The levels of BDNF were higher in males and females with ADHD compared to controls. However, the difference remained significant only in males with ADHD (Table 5).

**Table 1:** Comparison between age and sex distribution among the participants

	Group I	Group II	P
Age (years)	8.2 ± 2	8.6 ± 2.5	0.4$
Sex distribution (male percentage)	82.9%	75%	0.3^

$ t test; ^ chi-square test

**Table 2:** Results of the Stanford Binet intelligence scale subtests, the preschool language scale, and the Conner’s parent rating scale-R in children with ADHD

Items	Mean	SD	Items	Mean	SD
Fluid reasoning	98.75	10.35	Oppositional	69.6	13.3
Knowledge	92.78	8.88	Cognitive problems	74.8	10.5
Quantitative reasoning	99.5	10.14	Inattention/hyperactivity	64.8	17.8
Visuo-spatial	95.96	9.31	Anxious-shy	64.3	8.9
Working memory	93.25	12.57	Perfectionism	55	12.4
Non-verbal IQ	94.9	11.47	Social problems	70.1	15.4
Verbal IQ	97.71	10.57	Psychosomatic	69.8	12.2
Total IQ	96.8	11.78	Restless	70.7	17
Receptive language age (years)	5.76	1.42	Emotional liability	66.6	12.2
Expressive language age (years)	5.98	1.75	ADHD index	20.4	0.9
Total language age (years)	5.95	1.62	-	-	-

ADHD: attention deficit hyperactivity disorder; IQ: intelligence quotient; SD: standard deviation

**Table 3:** Comparison between the levels of expression of miRNAs and BDNF levels in children with ADHD and neurotypical children

Parameter	Group I (ADHD) Mean ± SEM	Group II (Neurotypicals) Mean ± SEM	P-value
miR-34c-3p	0.225 ± 0.036	1.016 ± 0.158	< 0.0001****
miR-155	1.010 ± 0.089	1.011 ± 0.108	0.7273
miR-138-1	0.462 ± 0.076	0.918 ± 0.107	0.0005**
miR-296-5p	0.745 ± 0.056	1.161 ± 0.165	0.1812
BDNF (ng/µl)	1455.676 ± 387.363	1018.605 ± 105.685	0.0006**

ADHD: attention deficit hyperactivity disorder; BDNF: brain-derived neurotrophic factor; miR: microRNA; ng: nanogram; µl: microliter; SEM: standard error of the mean;

*: *p* < 0.05; **: *p* < 0.01; ***: *p* < 0.001; ****: *p* < 0.0001; Mann-Whitney U test

**Table 4:** Gender differences in miRNAs expression profiles and plasma BDNF levels in ADHD and neurotypical groups

Parameter	ADHD Group			Neurotypical Group		
	Male (*n* = 34) Mean ± SEM	Female (*n* = 7) Mean ± SEM	P-value	Male (*n* = 30) Mean ± SEM	Female (*n* = 10) Mean ± SEM	P-value
miR-34c-3p	0.174 ± 0.032	0.344 ± 0.077	0.02*	1.026 ± 0.188	0.730 ± 0.183	0.6911
miR-155	0.916 ± 0.090	2.220 ± 0.398	0.0002***	1.208 ± 0.172	0.937 ± 0.179	0.6446
miR-138-1	0.458 ± 0.074	0.122 ± 0.054	0.1178	0.833 ± 0.096	1.878 ± 0.510	0.0703
miR-296-5p	0.770 ± 0.061	2.095 ± 1.002	0.9916	0.946 ± 0.114	5.158 ± 1.992	0.0726
BDNF (ng/µl)	1455.46 ± 331.7	1410 ± 648.6	0.4691	1056.75 ± 727	886.6 ± 113.801	0.3747

ADHD: attention deficit hyperactivity disorder; BDNF: brain-derived neurotrophic factor; miR: microRNA; ng: nanogram; µl: microliter; SEM: standard error of the mean;

*: *p* < 0.05 ***: *p* < 0.001; Mann-Whitney U test

**Table 5:** Gender comparison between ADHD and control groups regarding miRNAs expression and BDNF levels

Parameter	Males			Females		
	ADHD (*n* = 34) Mean ± SEM	Control (*n* = 30) Mean ± SEM	P-value	ADHD (*n* = 7) Mean ± SEM	Control (*n* = 10) Mean ± SEM	P-value
miR-34c-3p	0.174 ± 0.032	1.026 ± 0.188	< 0.00001 ***	0.344 ± 0.077	0.730 ± 0.183	0.02*
miR-155	0.916 ± 0.090	1.208 ± 0.172	0.2	2.220 ± 0.398	0.937 ± 0.179	0.009*
miR-138-1	0.458 ± 0.074	0.833 ± 0.096	0.0005***	0.122 ± 0.054	1.878 ± 0.510	0.005*
miR-296-5p	0.770 ± 0.061	0.946 ± 0.114	0.4	2.095 ± 1.002	5.158 ± 1.992	0.9
BDNF (ng/µl)	1455.46 ± 331.7	1056.75 ± 727	0.01*	1410 ± 648.6	886.6 ± 359	0.05

ADHD: attention deficit hyperactivity disorder; BDNF: brain-derived neurotrophic factor; miR: microRNA; ng: nanogram; µl: microliter; SEM: standard error of the mean;

*: *p* < 0.05 ***: *p* < 0.001; Mann-Whitney U test

## Discussion

ADHD is a common neurodevelopmental disorder, which interferes with an individual’s cognitive and language performance. Understanding the etiology and pathophysiology of this disorder, in addition to its possible influence on the aptitudes of children with ADHD, could offer attainable therapeutic targets for better intervention. It is necessary to explore patterns of expression of miRNAs in different populations, considering the complex nature of ADHD and the interaction between genes and environment regarding the development of its symptoms (Nuzziello et al. 2019).

In this study, memory abilities and knowledge were found to be the least developed among participants. These results indicated the disadvantages of this disorder for the cognitive abilities of children. These findings are in agreement with Kilany et al. (2022), who reported that working memory scores were the lowest among the IQ subitems. Nevertheless, it was the visuospatial ability, not the knowledge, which also revealed low scores in their sample. The memory problems could be related to the altered levels of BDNF, which was involved in memory consolidation. BDNF was reported to enhance neuronal growth and synaptic plasticity (Miranda et al. 2019). It has been involved in pre- and post-synaptic control of neurotransmitter release and regulation such as serotonin, glutamate, GABA, dopamine, and catecholamines (Colucci-D’Amato et al. 2020; Lima Giacobbo et al. 2019; Wang et al. 2022a). MiR-34 plays a critical role in memory formation, which is processed by the amygdala (Murphy and Singewald 2019). MiR-138 was found to control neuronal connections in the hippocampus in mice. The hippocampus has an essential role in memory consolidation (Daswani et al. 2022).

Receptive and expressive language skills in the current study were found to be delayed in children younger than 8 years old. The absence of discrepancies between receptive and expressive language ages in those with delayed language performance indicated that the language problems in these children were delays in development rather than a specific language impairment. The presence of language delays is in agreement with Hawkins et al. (2016), who reported that impairments in cognitive functions such as executive function interfere with language development in children with ADHD. On the other hand, basic language skills measured by the used language scale in children older than 8 years, including syntax and semantics, were not delayed. Goh et al. (2020) emphasized the relationship between early language problems and ADHD, which could be related to problems in some cognitive abilities in these children.

Considering that language and cognition are interrelated, alterations in essential neurobiological processes could impact both of them. The elevated levels of BDNF noticed in the ADHD participants of this study could have contributed to the deficits noticed in the abilities and behavior of participants with ADHD. High levels of BDNF could have led to deficits in the pruning of synapses responsible for the processing of memory and executive functions or to changes in the neurotransmitters’ levels, which were interrelated with BDNF and/or influenced by its action. Changes in synaptic quality influence connectivity between different brain areas involved in cognitive, language, and behavioral development (Liao et al. 2023). Increased interhemispheric connectivity was associated with reduced connectivity in the prefrontal cortex bilaterally and the right frontostriatal and frontoparietal neural networks (Chen et al. 2023). Furthermore, Yeom et al. (2016) reported that high BDNF levels had a negative influence on IQ and on indicators of behavioral problems in preschool children. The behavioral and cognitive problems in children with ADHD could also be related to changes in the levels of serotonin, glutamate, GABA, and dopamine, which were reported to be reduced in individuals with ADHD in previous reports, whereas levels of catecholamines were reported to be elevated. These neurotransmitters are essential for proper brain functioning (Banerjee and Nandagopal 2015; Dvořáková et al. 2007; Maltezos et al. 2014).

Heinrich et al. (2017) and Srivastav et al. (2018) depicted the regulatory role of miRNAs on BDNF gene expression. The downregulation of miR-138-1 could have led to overexpression of BDNF, increasing its level compared to controls. In the current study, we reported higher level of plasma BDNF in children with ADHD compared to their neurotypical peers with significant statistical difference. This finding aligned with previous studies, which targeted Egyptian children (El Ghamry et al. 2021) or other ethnic cohorts with ADHD (Gumus et al. 2022; Shim et al. 2008).

The reduced expression of miR-138-1 was in agreement with Wu et al. (2017), who reported downregulation of miR-138-1 in the prefrontal cortex of animal models with ADHD. They suggested that this downregulation was related to abnormalities in the miR-138-1 gene. This gene has been involved in visual perception processing. Interestingly, they discovered that the overexpression of the Nr3c1 gene was related to this miRNA downregulation. This gene has been linked to the hypothalamic-pituitary-adrenal axis and low cortisol levels, which were involved in ADHD development (Chen et al. 2019). The prefrontal cortex was among the brain areas that were reported to be altered in individuals with ADHD. This area was involved in attention and executive function control and processing (Wu et al. 2023). Martinez and Peplow (2024) reported that miR-138-1 expression levels were correlated with IQ scores, which could indicate its involvement in cognitive functioning. In this study, no correlations were detected between miR-138-1 and clinical measures. On the other hand, miR-138 was found by Zadehbagheri et al. (2019) to be upregulated in Iranian children with ADHD, which disagrees with the results of the present study. These differences in expression patterns highlight the importance of investigating miRNAs expression levels in different populations and in different gender distributions.

Alterations in the expression patterns of miR-34-c, either over or under the normal expression, would lead to perturbations in the processes that it is involved in. Abnormalities in this type of miRNA have been linked to polymorphisms in genes that were previously implicated in ADHD, such as BCL2, MET, and CREB1 (Garcia-Martínez et al. 2016). These genes were related to neuron development, neurotransmission, axonal growth, and cellular projection, as well as to lipid biosynthesis and metabolism. These genes were involved in central nervous system derangements and psychiatric disorders, including ADHD. Furthermore, miR-34-c is an essential component of the calcium-triggering mechanism, which aids neurotransmitter release in ADHD (Kim et al. 2014). The results of the downregulation of miR-34-c disagree with Garcia-Martínez et al. (2016), who investigated the level of miRNAs in a Spanish population and reported upregulation of this type of miRNA. Their study was conducted on adults with ADHD, and the males’ distribution was less than this study, which could also contribute to disagreement besides the population heterogeneity.

Correlation analysis in this study revealed that miR-34-c levels were negatively correlated with miR-138-1 levels. The detected correlation could stem from the opposing actions of these two miRNAs. Neuronal development is induced by miR-34, whereas apoptosis is induced by miR-138, which also inhibits proliferation. Despite having opposing actions, these two miRNAs were reported to be involved in memory processing. The importance of the proliferation of neurons and dendrites is as essential as their apoptosis. A proper balance between long-term potentiation and long-term depression is required for the optimal functioning of neurons and other brain cells. This balance contributes to memory processing and the proper development of other cognitive functions, including language integration, in developing brains (Stacho and Manahan-Vaughan 2022). The correlation results detected in the ADHD group were not identified in the control group.

The absence of significant differences in expression levels of miR-155 and miR-296-5p disagrees with Kandemir et al. (2014) and Wu et al. (2017), who reported significant differences between individuals with ADHD and neurotypicals in the form of upregulation of miR-155 and downregulation of miR-296-5p. This disagreement could be related to differences in the populations included, who were Chinese and Turkish. Nonetheless, a significant negative correlation was detected in the present study between miR-296-5p and the total IQ. MiR-296-5p was reported to regulate BDNF activity via Bhlhb2 gene suppression. This gene was reported to be involved in ADHD pathogenesis (Wu et al. 2017). It is worth noting that the expression of miRNAs is regulated by various factors, such as miRNA biogenesis, transcription factors, DNA copy number, and DNA methylation (Misiewicz-Krzeminska et al. 2019). This could explain the absence of correlations between the targeted miRNAs and other clinical measures in this study.

Some differences between males and females regarding the features of ADHD were reported. Females with ADHD exhibited more inattention problems compared to males (Graetz et al. 2005). However, few studies investigated the biochemical and miRNA levels using gender-based analysis. Szakats et al. (2023) explored the miRNA expression differences in males and females in animals. They detected gender differences in the patterns of expression in seven out of ten types of miRNAs they investigated, such as miR-206-3p, miR-200c-3p, and miR-205-5p. Although these types were not investigated in this study, the detected gender differences indicated the influence of biological sex on the expression of some types of miRNAs. Interestingly, the same pattern was noticed in the expression levels of the miRNAs and BDNF levels. Notwithstanding, the expression of miR-155 was found to be upregulated in females only with significant statistical differences. This is in agreement with Kandemir et al. (2014), who reported upregulation of this miRNA. However, they did not examine gender-based differences in their study. Wang et al. (2019) investigated the BDNF levels in children with ADHD, followed by a stratified gender analysis of these levels. The overall levels of BDNF did not differ from the control group. Nevertheless, the males with ADHD showed higher BDNF levels compared to controls, whereas the females showed lower BDNF levels. This is partially in agreement with this study considering the higher levels of BDNF detected in males and females with ADHD compared to controls, but they showed a significant statistical difference only in males. The differences between males and females could be attributed to hormonal differences, differences in polymorphisms of the targeted genes, or differences in the mRNA levels that miRNAs are controlling (Szakats et al. 2023; Wang et al. 2019). These findings highlight the importance of investigating gender differences in neurodevelopmental disorders.

The small sample size in this study could be considered a limitation. The stringent inclusion and exclusion criteria could have contributed to reducing the number of participants. Furthermore, the possible environmental factors, such as prenatal and perinatal histories, have not been compared between the groups. The participants were recruited from one clinic. However, it is a referral center for children with special needs, and it is frequently visited by participants from all Egyptian governorates. Despite these limitations, the inclusion and exclusion criteria were determined to eliminate factors that could influence the miRNA expression patterns. Moreover, this study is the first to investigate the miRNA expression patterns in Egyptian children with ADHD. The gender difference influenced the expression patterns in children with ADHD despite the low number of female participants in the ADHD group, which necessitates investigating this issue with a larger sample size in future studies. The expression levels of miR-34c-3p and miR-138-1, in addition to plasma BDNF concentrations, showed good discrimination accuracy between subjects with and without ADHD. Therefore, miR-34c-3p, miR-138-1, and BDNF can be candidate diagnostic biomarkers for ADHD.

## Conclusion

The downregulation of miR-34c-3p and miR-138-1 is suggested to be involved in the etiology of ADHD in Egyptian children. Both miRNAs, in addition to BDNF, can also be introduced as possible biomarkers for the diagnosis of ADHD. The expression levels of miR-296-5p may have an impact on the cognitive functions of children with ADHD. Considering that both miR-138-1 and miR-296-5p were involved in controlling BDNF levels, the BDNF level and its role in ADHD pathogenesis need further investigation. Interestingly, the gender difference in the included children with ADHD influenced the expression patterns of miRNAs within the ADHD group, but it did not show such influence in the control group. Furthermore, the gender difference influenced the discrepancy between cases and controls, which highlights the importance of investigating such differences in children with ADHD.

## Data Availability

Data and materials are available from the corresponding author on reasonable request.
